# Circumpapillary Course of Retinal Pigment Epithelium Can Be Fit to Sine Wave and Amplitude of Sine Wave Is Significantly Correlated with Ovality Ratio of Optic Disc

**DOI:** 10.1371/journal.pone.0122191

**Published:** 2015-04-07

**Authors:** Takehiro Yamashita, Taiji Sakamoto, Naoya Yoshihara, Hiroto Terasaki, Yuya Kii, Minoru Tanaka, Kumiko Nakao

**Affiliations:** Department of Ophthalmology, Kagoshima University Graduate School of Medical and Dental Sciences, Kagoshima, Japan; Saitama Medical University, JAPAN

## Abstract

The purpose of this study was to develop a method of quantifying the degree of optic disc tilt in normal eyes. This was a prospective, observational cross sectional study of 126 right eyes of 126 healthy volunteers. The optic disc tilt was determined from the circular peripapillary optical coherence tomographic (OCT) scan images. The course of the retinal pigment epithelium (RPE) layer in the peripapillary cross sectional scan images was fit to a sine wave curve, and the amplitude of the sine curve was used to reflect the degree of the optic disc tilt in the optical axis. The repeatability of the amplitude determinations was calculated. The correlation between the amplitude and the ovality ratio of the optic disc was determined. The correlation between the amplitude and the body height was also calculated. The mean amplitudewas 36.6 ± 17.5 pixels, which was significantly and inversely correlated with the ovality ratio of the optic disc (R = -0.59, *P*<0.001). The intra-rater and inter-rater correlation coefficients of the amplitude were significant high (*P*<0.001, both). The amplitude was significantly and inversely correlated with the body height (R = -0.38, *P*<0.001), but not with the axial length. In conclusion, a sine wave function can be used to describe the course of the RPE in the circumpapillary OCT images. The results indicate that the amplitude of the sine wave can be used to represent the degree of optic disc tilt. Thus, the sine wave analyses can be used as a quantifiable and repeatable method to determine the optic disc tilt.

## Introduction

Evidence has been obtained that myopia is a risk factor for primary open angle glaucoma (POAG), and several population-based studies have shown that individuals with myopia have a two- to three-fold higher risk of glaucoma than non-myopic individuals. [1–3] Considering the increasing prevalence of myopia worldwide, a correct diagnosis of glaucoma in myopic patients is becoming more important and necessary in ophthalmology. [1,4,5]

Optic disc tilt is one of the major changes in myopic eyes, and it is important to determine the degree of the tilt because it can affect visual functions. For example, healthy eyes with a greater optic disc tilt are generally associated with high myopia and reduced sensitivity on visual field tests.[6] Indeed, the presence of an optic disc tilt can affect the measurements of the mean deviations of visual fields and the peripapillary retinal nerve fiber layer (RNFL) thickness in glaucomatous eyes. [7,8]

There have been several investigations that evaluated the optic disc tilt quantitatively. Tay et al calculated the optic disc ovality by calculating the ratio of the minimum to maximum disc diameters measured in fundus photographs. [6] This analysis was based upon the hypothesis that the optic disc appears vertically longer in eyes in which the optic disc is tilted around a horizontal axis, and horizontally longer when the optic disc is tilted around a vertical axis. Additionally, the ovality ratio of the optic disc is influenced by the shape of the optic disc head which can make the measurements inaccurate in some cases. Takasaki et al examined eyes with horizontally and vertically tilted optic discs using the Heidelberg Retina Tomograph 3 printout and found that the degree of vertical tilt was closely related to the degree of myopia. [9] In addition, Hosseini et al and Hwang et al determined the horizontal and vertical optic disc tilts using spectral-domain optical coherence tomography (SD-OCT). [7,8] The former investigators evaluated the degree of optic disc tilt in the vertical or horizontal plane, and the latter did it in the foveo-optic disc axis plane. Although their methods were quantifiable, they did not determine the values in the plane where the optic disc was most tilted.

OCT is a noninvasive imaging method that can evaluate the morphology of the RNFL and optic disc with micrometer resolution. [10,11] Several studies have documented that OCT can obtain reliable measurements of the RNFL thickness. [12–17]

**Fig 1:**
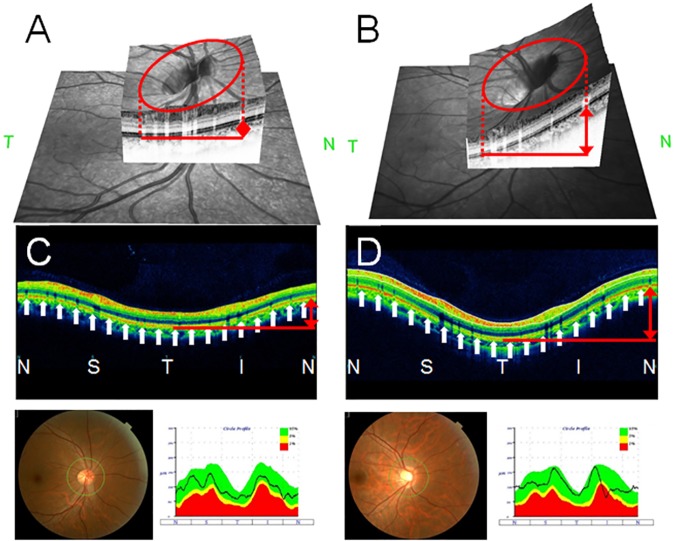
Three dimensional optic disc images and cross sectional images of the peripapillary retina. An eye with a smaller optic disc tilt (red arrow) and lower amplitude retinal wave (white arrows) is shown in A and C. An eye with a larger optic disc tilt (red arrow) and higher amplitude retinal wave (white arrows) is shown in B and D. Cross sectional images of the peripapillary retina, fundus photographs, and retinal nerve fiber layer thickness profiles of an eye with smaller optic disc tilt (C) and a larger (D) optic disc tilt.

Three dimensional optic disc images and cross sectional scan (B scan) images of the peripapillary retina of the RNFL circle scan are shown in Fig. 1. In this figure, both ends of the OCT images represent the nasal point of the optic disc and the center represents the temporal point. Because the optic disc usually tilts laterally, the retina is plotted at the upper coordinate on both ends and at the lower coordinate in the center.

We noted that the amplitude of the wave of the retina (Fig. 1, white arrows) became larger when the degree of the ovality or optic disc tilt became larger (Fig. 1, red up-and-down arrows). Careful examination of the shape of the wave showed that it resembled a sine wave. We then hypothesized that the amplitude of this retinal wave was significantly correlated with the degree of optic disc tilt. If this hypothesis is true, the degree of optic disc tilt can be quantified regardless of the direction of the optic disc tilt.

To test this hypothesis, we first determined a method to match the changes in the course of the retinal pigment epithelium layer around the optic disc to a sine wave. We then determined whether the amplitude of the sine wave was significantly correlated with the ovality ratio, a currently used method to quantify the degree of optic disc tilt. For further validation, we examined whether there was a significant correlation between the amplitude and the axial length of the eye and the body height because it has been reported that the optic disc tilt was correlated with the axial length of the eye and the body height. [18–20]

## Materials and Methods

All of the procedures conformed to the tenets of the Declaration of Helsinki. A written informed consent was obtained from all of the subjects after an explanation of the procedures to be used. The study was approved by the Ethics Committee of Kagoshima University Hospital, and it was registered with the University Hospital Medical Network (UMIN)-clinical trials registry. The registration title was, “Morphological analysis of the optic disc and the retinal nerve fiber in myopic eyes” and the registration number was UMIN000006040. A detailed protocol is available at https://upload.umin.ac.jp/cgi-open-bin/ctr/ctr.cgi?function=brows&action=brows&type=summary&recptno=R000007154&language=J. The results presented in this manuscript are part of an overall study. [21]

### Subjects

This was a cross sectional, prospective, observational study. We studied 133 eyes of 133 volunteers who were enrolled between November 1, 2010 and February 29, 2012. The volunteers had no known eye diseases as determined by examining their medical charts, and the data from only the right eyes were analyzed. The eligibility criteria were: age ≥20 years but ≤40 years; eyes normal by slit–lamp biomicroscopy, ophthalmoscopy, and OCT; best-corrected visual acuity (BCVA) ≤0.1 logarithm of the minimum angle of resolution (logMAR) units; and intraocular pressure (IOP) ≤21 mmHg. The exclusion criteria were: eyes with known ocular diseases such as glaucoma, staphyloma, and optic disc anomaly; presence of visual field defects; and history of refractive or intraocular surgery. Staphyloma was checked using B-mode echo and there was no staphyloma in this study participants. None of the eyes was excluded because of poor OCT image quality caused by poor fixation.

### Measurement of axial length and refractive error

All of the eyes had a standard ocular examination including: slit-lamp biomicroscopy of the anterior segment; ophthalmoscopy of the ocular fundus; IOP measurements with a pneumotonometer (CT-80, Topcon, Tokyo, Japan); and axial length measurements with the AL-2000 ultrasound instrument (TOMEY, Nagoya, Japan). The refractive error (spherical equivalent) was measured with the Topcon KR8800 autorefractometer/keratometer.

### Determination of amplitude and ovality ratio

All eyes were examined by a single examiner (TY). The peripapillary region was measured with the Topcon 3D OCT-1000 MARK II using the RNFL 3.4 mm circle scan for the RNFL thickness. In this protocol, 1024 A-scans/circle, and 16 overlapping B-scans/image and direct B-scan recordings were made. The OCT images and the color fundus photographs were taken at the same time. Great care was taken to maintain the facial frontal plane vertical to the reference light axis and to position the camera on the central axis because the position can affect the curvature in the B-scan images. During the measurements, the examinee was asked to fixate a reference light. The OCT system detected the edge of the optic disc, and the scan circle was automatically centered on the optic disc before recording the OCT images. To exclude the effects of errors in the centering of the scan circle, one examiner (YK) checked that the center of the scan circle was located at the center of the optic disc offline.

**Fig 2:**
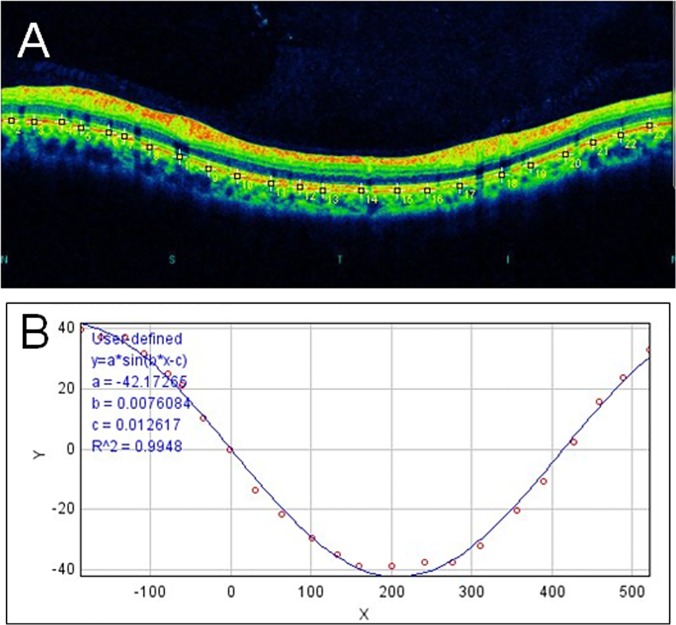
Optical coherence tomography of circumpapillary retinal layer and its converted coordinates to sine curve equation. A: The locations of the retinal pigment epithelium are marked on the retinal nerve fiber layer B-scan image (yellow squares) by two masked examiners. The pixel coordinates of each position were determined automatically using ImageJ and converted to a new set of data with the center of the wave as the origin. B: The converted coordinates were fit to a user-defined sine curve equation, *y* = *a* × sin(b×x-c), using the curve fitting program of ImageJ.

The course of the retinal pigment epithelium (RPE) were plotted on the B-scan images manually (Fig. 2A). The coordinates of each pixel were determined automatically using the ImageJ program (Image J version 1.47, National Institutes of Health, Bethesda, MD, USA; http://imagej.nih.gov/ij/ [in the public domain]). The ‘x’ and ‘y’ coordinates of the B-scan images were converted to a new set of ‘x’ and ‘y’ coordinates with the center of the wave as the origin. Finally, the converted data were fit to a sine wave equation (*y* = *a* × sin(b×x-c)) with the curve fitting program of ImageJ (Fig. 2B). The ‘a’, ‘b’, and ‘c’ are constants calculated by the least squares method of curve fitting of ImageJ. The constant ‘a’ is the amplitude of the sine curve, and a larger amplitude ‘a’ will make the amplitude of the curve larger and make the arms of the optic disc tilt more. Because the eye was stationary throughout the measurements, the retinal plane of macular area was constantly approximately perpendicular to the reference light axis. Thus, the optic disc tilt examined in this study is assumed to show the angle between the optical axis and the optic disc tilt correctly. The amplitude of the sine wave, ‘a’, was considered to reflect the degree of the optic disc tilt against the optical axis.

To validate this method, the amplitude were compared to a commonly used method, the ovality ratio. The ovality ratio was determined with modification from the color fundus photographs as described in detail. [6] The maximum and minimum disc diameters were measured by a single observer using the Photoshop software. We defined a vertically long disc as that in which the axis of longest diameter was less than 45 degrees and the horizontally long disc as that in which the axis of longest diameter was more than 45 degree in a clockwise manner. If the optic disc was vertically longer, the ovality ratio was calculated from the ratio of the minimum divided by the maximum disc diameter. If the optic disc was horizontally long, the ovality ratio was calculated as the ratio of the maximum divided by the minimum disc diameters.

### Statistical Analyses

All statistical analysis were performed with the SPSS statistics 19 for Windows (SPSS Inc., IBM, Somers, New York, USA). Because the course of the RPE was plotted manually, the intra-rater or the inter-rater correlation coefficients of the amplitude were calculated using a two-way mixed-effects model for measurements of absolute agreement. For intra-rater comparisons, each RPE layer was plotted two times independently by a single rater (NY) with no information of eyes or the results by the other examination. For inter-rater comparisons, each image was measured by two independent raters (NY, HT) with no information of eyes or the results by the other rater. The relationships between the refractive error, axial length, ovality ratio, body height, and the amplitude were determined by Spearman’s correlation analyses.

## Results

One hundred and thirty-three Japanese volunteers were studied. Seven eyes were excluded due to ocular diseases or prior ocular surgery; three cases because of superior segmental optic hypoplasia, one case because of glaucoma, and three cases because of laser-assisted *in situ* keratomileusis. In the end, the right eyes of 126 individuals (85 men and 41 women) were used for the analysis.

The demographic information of the volunteers is presented in Table 1. The mean ± standard deviation of the age was 26.0 ± 4.1 years, and the mean refractive error (spherical equivalent) was -4.71 ± 3.41 diopters (D). The mean axial length was 25.4 ± 1.4 mm. The refractive errors and axial lengths were significantly and negatively correlated (R = -0.82, *P* <0.001).

**Table 1:** Participants data.

	Mean ± SD	Range
**Age (years)**	26.0 ± 4.1	22 ~ 40
**Sex (M/F)**	85 / 41	
**Spherical equivalent (diopters)**	-4.71 ± 3.41	-14.25 ~ 4.50
**Axial length (mm)**	25.4 ± 1.5	22.3 ~ 30.4
**Ovality ratio**	0.89 ± 0.11	0.60 ~ 1.42
**Body height (cm)**	167.6 ± 8.4	149.5 ~ 185.5
**Amplitude (pixels)**	37.0 ± 17.5	4.8 ~ 80.8

SD: standard deviation

### Intra-rater and inter-rater repeatability of determination of amplitude

The intra-rater and inter-rater repeatability of the amplitude measurements was determined for the right eyes of the 126 participants. The intra-rater correlation coefficients of the amplitude was 0.996 (95% confidence interval (CI) 0.994 to 0.997, *P* <0.001). The inter-rater correlation coefficient of the amplitude was 0.959 (95% CI 0.942 to 0.971; *P* <0.001). The mean and standard deviation of the amplitude was 37.0 ± 17.5 pixels. Because the intra-rater and the inter-rater repeatability of the amplitude were excellent, the means of the amplitude of the two raters were used for the analyses.

### Spearman’s correlation coefficients between amplitude and ovality ratio, refractive error, axial length, and body height

**Fig 3:**
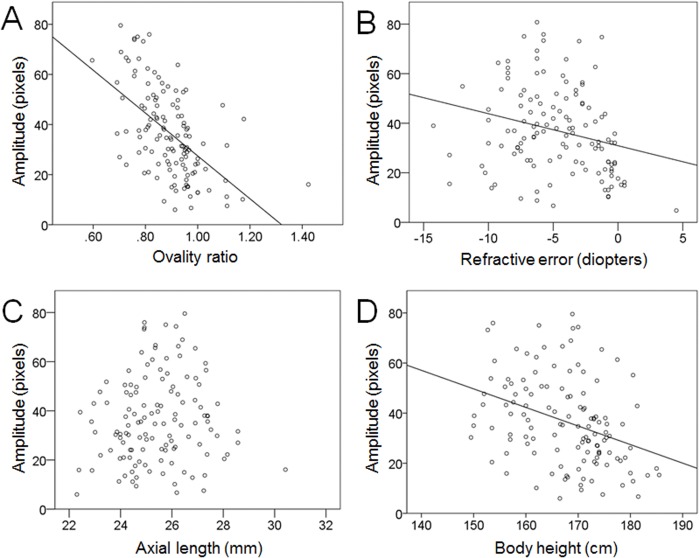
Scatterplots of amplitude. Plots of the ovality ratio (A), refractive error (spherical equivalent) (B), axial length (C), and body height (D).

The amplitude of the sine wave, i.e., the optic disc tilt, was significantly and inversely correlated with the ovality ratio (R = -0.59, *P* <0.001). The amplitude was also significantly and inversely correlated with the refractive error (spherical equivalent; R = -0.29, *P* = 0.001) but not with the axial length (R = 0.11, *P* = 0.21). The amplitude was significantly and inversely correlated with the body height (R = -0.39, *P* <0.001; Fig. 3).

## Discussion

We have developed a new method to quantify the degree of optic disc tilt from the SD-OCT circle scan images using a mathematical function. The coefficient of determinant of the curve fitting was greater than 0.90 in 92.1% (116/126) of the cases, which means that the sine wave equation was a good fit for the optic disc tilt. Additionally, the repeatability was excellent.

There have been several studies that have quantified the degree of optic disc tilt. The effect of an elongation of the axial length or refractive error of the eye can empirically be most evident on the X-Y axis of the disc diameter, i.e., horizontal changes. In the original method by Tay et al, the ovality ratio was always calculated as ratio of shortest diameter divided by longest diameter (shortest diameter/longest diameter). [6] Thus, the ovality ratio of horizontally elongated disc reflected the effect along the X-Z axis, vertical, rather than horizontal changes. If the discs of these two types would be evaluated together with no distinction, the effect of elongation of axial length would be underestimated.

Therefore, we modified the method of determining the ovality ratio. We defined the vertically long disc as that in which the axis of longest diameter was less than 45 degrees and the horizontally long disc as that in which the axis of longest diameter was more than 45 degree in a clockwise manner. In the eyes with vertically long axis (vertical axis group), the ovality ratio was calculated by dividing shortest diameter by longest diameter as Tay’s original method. While, in the eyes with horizontally long axis (horizontal axis group), the ovality ratio was calculated by dividing longest diameter by shortest diameter, modified ovality ratio. Consequently, the ovality ratio of eyes with a vertically long disc was less than 1.0 and that of eyes with a horizontally long disc was greater than 1.0. Our results showed that 115 eyes were included in vertical axis group. In this group, there was a significantly negative correlation between the ovality ratio and the amplitude (R = -0.58, *P* <0.001), which was almost the same as the results of all eyes (Fig. 3A). There were 11 eyes in the horizontal axis group, however, the modified ovality ratio was not significantly correlated with the amplitude (R = -0.29、*P* = 0.39). We could not figure out the reason of this results, but the disc with horizontal axis might better be evaluated separately to see the effect of axial length. Another study with more numerous cases would be necessary to find the reason.

Takasaki et al assessed the degree of horizontal and vertical optic disc tilt using the Heidelberg Retina Tomograph 3 printout. [9] The degree of horizontal tilt was taken to be the angle between a horizontal line and a line that was drawn manually to connect the two points where the height profile and the disc margin met. The degree of vertical tilt was determined in a similar manner. However, the horizontal line in the height profile could be affected by rotations and torsions of the optic disc. Additionally, the manually-drawn line connecting the disc margins in the height profile can be affected by a manually-drawn contour line. In addition, these horizontal and vertical cross sectional images did not necessarily detect the most tilted direction of the optic disc because most tilted direction could be on an oblique line and not on a horizontal or vertical line.

Hosseini et al determined the vertical tilt angle on the SD-OCT image. [7] On the SD-OCT image in the vertical plane of the optic disc, the line between the nasal edge of optic disc and the nasal edge of RPE line temporal to the optic disc was used as a reference line. The optic disc plane was defined as the line between the nasal and temporal edge of the optic disc in the same OCT image. The angle formed by these lines was used as the degree of optic disc tilt. This was a reasonable approach, however the results were strongly affected by the presence of a conus. The vertical tilt angle method quantifies the discrepancy between the edge of RPE line and optic disc edge. This discrepancy generally coincides with the width of conus. As a result, the larger the conus is, the greater the optic disc tilt becomes by this method. However, even if an optic disc tilt exists, it can be expressed as no optic disc tilt by this method. Indeed, there are many eyes with optic disc tilt but no conus, and the vertical tilt angle method measures the extent of the conus and not the optic disc tilt. Additionally, this method can evaluate the angle of optic disc tilt only around the fovea-disc axis plane.

It is widely accepted that the optic disc tilt increases as the axial length increases. [6–9] However, no significant correlation was found between the amplitude and the axial length in our subjects. This could be because our cases were skewed toward myopia, i.e., the average refractive error was -4.71 ± 3.41 diopter which is more myopic than that of the eyes of earlier studies. [6–9] Our cases were Japanese students volunteers who are known to belong to one of the most myopic groups in the world. Thus, our results might not necessarily hold for non-myopic populations. An epidemiological study should help generalize the present results to all populations.

There are two major growth patterns of the eyeball. One is a proportional enlargement where an eyeball becomes spherical. The other is an excessive enlargement in the antero-posterior axis, where the eye becomes more elliptical. [22–24] The optic disc may not tilt in the former pattern, but it can tilt in the latter pattern. This may explain why the optic disc tilt can vary in different eye with the same axial length. In future study, the shape of the eye needs to be evaluated to study the optic disc tilt.

There are strengths in this study. Compared to the earlier methods, the present method is better because the optic disc tilt in the optical axis can be quantified regardless of the direction of optic disc tilt. The examination requires only the standard clinical RNFL scan protocol which is incorporated in most OCT instruments. In addition, other scanning protocols and a special software are not necessary. Above all, the analysis can be easily done by the publically accessible software ImageJ. These will allow many other researchers or clinicians to repeat and evaluate the present method.

There were also limitations of this study. There are many factors which can affect the RNFL circle scans, e.g., the size, shape, and torsion of the eye. [25–27] None of these were considered in our analyses. The scan circle is projected larger than the actual circle in eyes with a longer axial length by the magnification effect. This might affect the RPE curve. Unfortunately, it may not be possible to adjust the same scan circle diameter corrected for axial length for our OCT instrument. Only the effect of the eye torsion could be minimized by use of the sine wave approach. The coefficient of determinant of the curve fitting was not greater than 0.90 in only 7.9% (10/126) of the cases. The RPE curve of these cases was almost flat. In such cases, a rectilinear RPE line did not fit the sine curve equation. However, even in such cases, the optic disc tilt became small, indicating that this method could work with these rectilinear RPE line.

In summary, we were able to use a mathematical method to fit a sine wave to the curvature of the RPE in the circular OCT scan images. The coefficient of determinant of the curve fitting was greater than 0.90 in 92% of the scans. Most important was that there was a highly significant correlation between the amplitude of the sine wave, and the ovality ratio, a commonly used method to quantify the degree of the optic disc tilt. We conclude that the amplitude of the sine wave can be used to represent the degree of optic disc tilt. This method is simple and easy to perform and requires only a B-scan image of the RNFL thickness and an easily accessible free public software. This type of analyses should be of great value for clinicians and researchers to study the pathophysiology of the optic disc.

## Supporting Information

S1 Dataset Dataset. (XLSX)
